# Toolkitting: an unrecognized form of expertise for overcoming fragmentation in inter- and transdisciplinarity

**DOI:** 10.1057/s41599-024-03279-9

**Published:** 2024-06-29

**Authors:** Bethany Laursen, Bianca Vienni-Baptista, Gabriele Bammer, Antonietta Di Giulio, Theres Paulsen, Melissa Robson-Williams, Sibylle Studer

**Affiliations:** 1 Michigan Institute for Clinical & Health Research, University of Michigan, Ann Arbor, MI, USA.; 2 Laursen Evaluation & Design, LLC, Grand Rapids, MI, USA.; 3 Cultural Studies of Science and Technology Group (CSTS); Transdisciplinarity Lab, Swiss Federal Institute of Technology, ETH Zurich, Zurich, Switzerland.; 4 National Centre for Epidemiology and Population Health, The Australian National University, Canberra, ACT, Australia.; 5 Department of Social Sciences; Department of Environmental Sciences, University of Basel, Basel, Switzerland.; 6 Network for Transdisciplinary Research (td-net), Swiss Academies of Arts and Sciences, Bern, Switzerland.; 7 Manaaki Whenua Landcare Research, Lincoln, Aotearoa New Zealand.

## Abstract

A growing number of inter- and transdisciplinary (ITD) toolkits provide methods, processes, concepts, heuristics, frameworks, and other resources for designing and implementing ITD research. A brief overview of the currently fragmented toolkits landscape is provided, fleshed out through descriptions of four toolkits. Fragmentation means that researchers are unaware of, and do not have access to, the full array of tools that could benefit their investigations. Overcoming fragmentation requires attention to toolkitting, which is the relatively overlooked bundle of practices involved in the creation, use, maintenance, funding, and study of toolkits. In particular, the processes and expertise involved in the creation, maintenance, and study of toolkits are described. Toolkitting as metawork can make resources more accessible, useful, and rigorous, enhancing ITD research. Future toolkitting can be strengthened with attention to key questions that can guide the activities of, respectively, toolkit creators and curators, scholars, and funders. Examining the toolkits landscape through the lens of toolkitting suggests that the development of a comprehensive, ongoing inventory is a first step in overcoming toolkit fragmentation. An inventory could also be the foundation for an even bolder initiative—a federated knowledge bank—that connects and develops the range of existing and future toolkits. The inventory and federated knowledge bank also provide a shared project to bring together the expertise of ITD toolkit creators, curators, users, funders, and scholars to achieve a step-change in enhancing ITD research.

## Introduction

While the globe suffers from complex problems, those tackling such problems using inter- and transdisciplinary (ITD) research must often spend precious time and effort re-learning, re-creating, and re-legitimizing promising practices across projects. Unlike disciplinary research, inter- and transdisciplinary research has no established canons of tested and well-referenced methods and other tools. Instead, knowledge about ITD tools is often tacit and, when published, is scattered across a wide range of literature (Defila and Di Giulio 2015; Bammer et al. 2020; Vienni-Baptista et al. 2020). This fragmentation makes it hard to find, use, and justify tools appropriately, slowing ITD projects and delaying any transformative outcomes they might deliver.

To overcome this fragmentation, many organizations and individuals have created ITD toolkits. As curated collections of resources, toolkits provide methods, processes, concepts, heuristics, frameworks, and other assets for designing and implementing ITD research. However, ITD toolkits are now suffering from the same fragmentation facing ITD tools themselves, with multiple toolkits created by different communities without awareness of each other’s work. There is thus a pressing need to address how toolkits are created and sustained. In the absence of such action, ITD efforts will likely fail to reach their potential.

We write as core members of the Toolkits and Methods Working Group hosted within the Global Alliance for Inter- and Transdisciplinarity (ITD Alliance; https://itd-alliance.org/working-groups/toolkits_methods/). Since 2020, we have jointly mapped and visualized the previously uncharted landscape of ITD toolkits. Individually, we have variously (co-)created ITD toolkits, studied them, used their contents, and leveraged them to build new fields of scholarship and practice. This article is based on our individual and collective analysis, reflections, and experience, primarily as researchers, with all the corresponding limitations. Nevertheless, we are not aware of any other in-depth examination of how toolkit work unfolds.

In this article we do not address a specific research question; instead, we use our review and reflections to spotlight the unrecognized expertise involved in the creation, maintenance, and study of toolkits—three practices in a bundle we call toolkitting. Toolkitting involves a unique form of ITD expertise, distinct from the research expertise toolkits themselves aim to support. With strategic development, toolkitting expertise can build the ITD field and unleash the potential of ITD toolkits.

We begin by briefly reviewing the ITD toolkits landscape and detailing four toolkits that some of the authors have been involved in developing. We then examine the expertise required for toolkitting, focusing on toolkit creation and maintenance, as well as the study of toolkits and toolkitting. Finally, we conclude with questions for toolkit creators, curators, funders, and scholars to guide the future of toolkitting, culminating in proposed inventory and federated knowledge bank projects to overcome the problem of fragmentation.

## Toolkits that support ITD expertise

Without attempting to be exhaustive, our Working Group identified 64 English-language toolkits relevant to inter- and transdisciplinarity, as well as others in German, Spanish, Dutch, and Japanese (https://itd-alliance.org/working-groups/toolkits_methods/inventory-project/). As we describe in more detail below, some toolkits explicitly focus on ITD research, a few focus more broadly, and several focus on specific aspects of ITD research organized by theme or problem domain.

Four noteworthy early examples are:

1. The Integration and Implementation Sciences (i2S) resources repository (started in 2002 and decommissioned in 2023, merging into i2Insights – see Table 1), which had a broader remit than inter- and transdisciplinarity (Bammer and Deane 2002-onwards),
2. Catherine Lyall’s “Interdisciplinary Wiki”, which started in 2009 to document a workshop series and has since evolved (Lyall 2009–onwards),
3. Rick Szostak’s “About Interdisciplinarity” sponsored by the Association for Interdisciplinary Studies (Szostak 2013–onwards), and
4. A book compiling integration methods for transdisciplinary research edited by Matthias Bergmann and colleagues, published in German in 2010 and in English in 2012 (Bergmann et al. 2012). This example also highlights the importance of textbooks as toolkits.

Other toolkits focus on particular practices relevant to ITD research. Examples of these cross-cutting themes and their toolkits include:

1. Teamwork—The Team Science Toolkit (started in 2008 and removed in 2022; National Institutes of Health National Cancer Institute 2008) and the Team Science Community Toolkit (COALESCE 2023),
2. Stakeholder engagement—*BiodivERsA’s Stakeholder Engagement Handbook* (Durham et al. 2014), and
3. Dialogue methods—*Research Integration Using Dialogue Methods* (McDonald et al. 2009).

Still other toolkits are motivated by a particular real-world problem; these are often related to socio-environmental sustainability (e.g. Will Allen’s Learning for Sustainability website started in 2005 and the STEPS Center’s Pathways to Sustainability methods toolkit from 2021).

To describe what toolkits and toolkitting involve beyond these brief examples, we next detail four toolkits developed by members of our Working Group. Table 1 summarizes key features of these four toolkits, and this is followed by a brief description of each toolkit. Although this is a convenience sample, the in-depth experience of establishing and maintaining these toolkits has provided rich lessons that significantly informed the examination of expertise presented in this article.

These four toolkits vary in focus and size:

- td-net Toolbox
  The td-net Toolbox focuses on the co-production of knowledge in heterogeneous groups, bringing together science and practice. It provides an alphabetical set of 20 methods with detailed instructions co-written with tool authors, while another ten are characterized by td-net personnel and linked to external sources. Tools are searchable by process phase and key issues, streamlining their selection for tasks such as project planning and addressing cross-cutting challenges. A “shared experiences” section shows researchers adapting tools to their contexts and reflecting on lessons learned and future method development. Emphasizing a focused selection over a broad range, the td-net Toolbox is designed for efficient method selection, enriched with context and references in the “Thoughtful Use of td Methods and Tools” section.
- Integration and Implementation Insights (i2Insights) blog and repository
  The focus is on tools for tackling complex societal and environmental problems, hence the toolkit draws on not only accepted transdisciplinary and interdisciplinary tools, but also those from systems thinking, action research, implementation science, post-normal science, and more. It differs from most other toolkits by inviting contributions from any researcher, educator, or practitioner tackling complex problems, with the aim of building a global community as well as a repository of resources. In general, a new tool is added every week, and tools are available as a date-ordered blog scroll (more than 500 tools as of this writing). Each tool is indexed using 14 main topics (e.g., diversity and integration), 11 resource types (e.g., method and framework), author name, and several hundred descriptive tags.
- Integrated Research Toolkit
  The focus is on assisting researchers who are new to integrative and cross-knowledge system research. The toolkit contains simple resources like tools and methods for designing and carrying out research, and working in teams, with stakeholders, and with Māori (Aotearoa New Zealand’s Indigenous people). It contains curated guides for beginners about specific aspects of integrated research, such as developing integrated research bids or deciding who to involve. The resources in the Integrated Research Toolkit have been drawn from a range of sources across inter- and transdisciplinarity, systems thinking, and collaborative practice as well as from sources for working with Māori. Each is communicated such that it is accessible to beginners. The toolkit continues to grow, and it currently contains 56 resources presented in a range of ways to help researchers find the right tool or method. Resources are arranged alphabetically, by research phase or topic, and within guides and case studies to show how specific tools have been employed in practice. Each resource page contains a section directing users to similar or related tools.
- SHAPE-ID Toolkit
  The focus is to signpost best practices for interdisciplinary and transdisciplinary research that integrates Arts, Humanities, and Social Sciences alongside societal partners and researchers from the Sciences, Technology, Engineering, and Mathematics. Tools are organized around nine topics related to ITD research, including understanding inter- and transdisciplinarity, co-designing research conditions for collaborating, developing ITD research skills, funding collaborative research, and evaluating ITD projects. The five kinds of resources are: (1) short case studies and video clips; (2) “Top Ten Tips”; (3) collaborative tools, (4) fact sheets, and (5) guided pathways to resources. Unlike the other three toolkits, this one is linked to a specific project, as described in the table above. The toolkit is accompanied by a volume that follows the same structure and provides further resources (Vienni-Baptista et al. 2023).

Even with only four detailed examples explicitly related to inter- and transdisciplinarity, it is clear that toolkits diverge in their purposes, contents, and the types of expertise they aim to help build. Moreover, there are many more toolkits that do not reference “inter- and transdisciplinarity” but are nevertheless relevant, such as toolkits in design or systems thinking. These support an even greater range of skills and knowledge helpful for conducting ITD research (Studer and Pohl 2023).

But beyond the target expertise that they seek to help build, ITD toolkits also represent another form of expertise previously unrecognized and underdeveloped in inter- and transdisciplinarity—the expertise of toolkitting.

## Toolkitting as ITD expertise

*Toolkitting* is a bundle of practices that includes the creation, use, maintenance, funding, and study of toolkits. While toolkits—the products—receive much attention, toolkitting—the process—has been relatively overlooked both as a leverage point for action and as a subject of study in its own right.

Toolkitting expertise is distinct from ITD expertise and builds the ITD field by guiding the process of toolkitting into constructive directions. Toolkitting is a form of metawork, or work that organizes work (Gerson 2013; van der Tuin 2021). That is, toolkitting is a different kind and level of work that systematizes how ITD research is done on the ground. Such metawork can be powerful, especially when it impacts the research process and channels funding and policy making. Toolkitting is a way of canonizing knowledge that has been in flux or shared mainly through highly contextual, interpersonal learning, often based on tacit knowledge. As Ankeny and Leonelli (2016) would put it, toolkits are keystones in the emerging “repertoires” that establish new fields and consolidate emerging practices.

Seen in this way, toolkitting has major benefits in making ITD knowledge available to people beyond the originating community, giving them more options for understanding and addressing their challenges. Toolkitting can enlarge the user community, galvanizing broader collective action and enabling further innovations (O’Rourke 2017; Bammer et al. 2020). Collecting ITD expertise in a recognized container like a toolkit can help legitimize that expertise and therefore bolster the security and confidence of the toolkit users (Bammer 2014). In doing so, toolkits can begin to create standards for good practice that raise expectations and the overall quality of ITD research (Defila and Di Giulio 2015). Working through toolkits may, in fact, be an ideal way to conduct ITD research and to build the ITD field: toolkits provide just enough structure to get collaborations off the ground while allowing (requiring) users to choose which tools to use and how to adapt them to local conditions (van der Tuin 2021).

As Table 1 illustrates, typically any attention to the process of toolkitting has been limited to a specific toolkit’s rationale, content, structure, and acknowledgment of funding. Only rarely is information provided about how the toolkit is used and maintained. Further, we are aware of only one essay addressing how ITD toolkitting can be studied, with a narrower focus than the one we elaborate in this article (van der Tuin 2021). Seeing these lacunae, our hard-won experience has motivated us to systematize the expertise needed to toolkit better. Next, we articulate key aspects of *what* is done in several types of toolkitting (the process) and *how* it is done well (the expertise). Much of our experience has been gained as toolkit creators, curators, and scholars, so that is where we focus here. Toolkit use and funding remain important areas for future elaboration, as we indicate in section 4. We reflect on the limitations of our approach in the conclusion.

### Toolkit creation

#### Process.

In principle, toolkit creation involves at least four phases: definition, design, development, and dissemination, although in practice these may be indistinct, intertwined, and incomplete. These phases are assisted by establishing a governance structure to guide how decisions are made and enacted, as well as ensuring that there is sufficient capability and capacity to create the toolkit —although again, in practice, the governance structure may not be articulated or transparent.

The process of toolkit creation begins with defining a vision or rationale for why a toolkit is needed. For example, the td-net Toolbox is part of a vision which, together with the td-net network and the ITD Conference, aims to foster transdisciplinarity in Switzerland and abroad. The Integrated Research Toolkit aims primarily to meet needs expressed by researchers who are new to inter- and transdisciplinarity. In such a vast space as inter- and transdisciplinarity, and with the growing number of toolkits, it is important for toolkits to clarify which theoretical approach and user community they aim to support.

Toolkit design tasks include choosing the structure and content. Methods from design research, or at the very least a user-centered perspective, are especially apt in this stage to ensure the toolkit will meet user needs. When toolkit creators also have experience in designing and conducting the type of ITD research that the toolkit aims to support, it is generally easier to make user needs central.

In terms of structure, the Shape-ID Toolkit, the td-net Toolbox, and the Integrated Research Toolkit are all structured around various goals users might want to achieve, such as understand inter- and transdisciplinarity or evaluate ITD projects. These three toolkits, in turn, provide multiple ways for users to navigate this structure. For example, the SHAPE-ID Toolkit identified four user journey types and included these as “guided pathways” for exploring the toolkit. Both the td-net Toolbox and Integrated Research Toolkit offer guides based on phases of a project. In contrast, the i2Insights blog and repository uses an index to structure the toolkit and has published two primers as guides to the topics of understanding diversity and stakeholder engagement. O’Rourke (2017) notes that while it is possible to organize a toolkit based primarily on theory (e.g., Bammer 2014), taking into account how users view the tools is likely to increase use and to capture innovations in the field more quickly.

Regarding content, the tools included can range widely, as in the i2Insights blog and repository, the Integrated Research Toolkit, and the Shape-ID Toolkit, or they can be restricted to particular topics such as knowledge co-production for the td-net Toolbox or stakeholder engagement in toolkits such as that provided by Durham and colleagues (2014). Toolkits may be a one-time creation, or they may be constantly growing and evolving as are the four toolkits we detailed above.

Designing the structure and content also entails designing the material platform of the toolkit. This nearly always requires collaborating with technical specialists who may not have expertise in inter- and transdisciplinarity. Toolkits produced as books require work with publishing specialists such as editors, while online toolkits may involve working with database designers and web developers. When choosing the platform, many considerations come into play, including availability, ease of use, aimed-for longevity, and transferability should circumstances change. The td-net Toolbox opted to host their toolkit on the website of the Swiss Academy of Sciences, which was not only prestigious, but also ensured the toolkit would remain available long term. The i2Insights blog and repository is built on the WordPress platform, which is relatively inexpensive and easy to use. As a third example, the Shape-ID toolkit was designed by a sub-contracted company.

Next, developing the toolkit essentially involves collecting the contents and placing them into the toolkit structure. Here the relationship with technical specialists such as web developers tightens. For some toolkits, relationships with ITD users and contributors also blossom in this phase. For example, the i2Inisghts blog and repository relies on existing relationships, as well as new connections, to recruit a steady stream of contributions and comments. When placing contents into the toolkit structure, creators can increase findability, accessibility, interoperability, and reusability of the contents by applying keywords and other metadata to the contents. To this end, the i2Insights blog and repository accepted the invitation to register its keyword ontology in BioPortal, a global repository of ontologies.

Finally, dissemination tasks include marketing the toolkit, onboarding initial users, and expanding the user base. These tasks can be pursued using greater or lesser degrees of collaboration with end users. Where there is collaboration with end users, a toolkit has a head start in the dissemination process, as at least some of the target audience will know about it. For example, the td-net Toolbox and Integrated Research Toolkit originally targeted educators and/or researchers in their home institutions and they were involved in its development. Nevertheless, toolkits will almost always aim for a broader audience than those originally consulted, with dissemination avenues including social media and other forms of networking. Academic publications have also been used to announce the availability of toolkits (e.g., Vogel et al. 2013; Bammer 2015; Studer and Pohl 2023).

All phases of toolkit creation benefit from a governance or decision-making structure. This structure can range from a board or advisory group, as is the case for the td-net Toolbox and the Shape-ID Toolkit, to decision-making by the founder, as with the i2Insights blog and repository and the Integrated Research Toolkit. The decision makers also need to ensure sufficient capability (described under *Expertise* below) and capacity (person-power) to undertake all the relevant toolkitting tasks.

#### Expertise.

In this section, we list aspects of relevant expertise with brief explanations as a series of dot points. We begin with expertise required in all phases of creating a toolkit, followed by expertise roughly divided by each phase.

All phases:

- Familiarity with inter- and trans-disciplinarity as a topic and a culture. Ideally this involves broad expertise in conducting or accompanying ITD research that covers an array of topics. It is noteworthy, though, that building a toolkit is also a learning exercise.
- Project management skills, especially keeping track of tasks throughout all phases.
- Relational expertise, especially expectation management in being able to work with tool contributors, toolkit users, and toolkit teammates such as project managers and web developers.

Defining the toolkit’s rationale:

- Assessment skill to compare what users need with what already exists.
- Strategy selection skill to identify what it is feasible to offer to meet user needs.
- Theoretical awareness to select a clear definition of the sort of ITD work the toolkit aims to support.

Designing the toolkit:

- Information schema selection skills for organizing the toolkit’s contents.
- Familiarity with user experience and user interface research methods.
- Knowledge about platform affordances for selecting a platform to support the toolkit’s aims.

Developing the toolkit:

- Discernment for deciding what is not only relevant but high enough quality to be included, plus awareness of one’s own biases in making these decisions.
- Search and/or networking skills to find the resources to be compiled into the toolkit.
- Technical skills in developing the chosen platform (e.g., book, website).
- Communication skills to make the unique value of each tool as understandable as possible.
- Indexing skills to aid in tool findability, accessibility, interoperability, and reusability.

Disseminating the toolkit:

- Marketing and advertising skills to communicate the unique value of the toolkit clearly and broadly to potential users.
- Onboarding process design skills to craft a welcoming onramp for new users.

### Toolkit maintenance

#### Process.

Toolkit maintenance involves keeping links to tools functional, replacing or updating outdated tools, and adding new tools. Toolkits produced by one-time project funding, rather than ongoing funding, generally do not have the resources to undertake any maintenance. Similarly, toolkits produced as books are generally not updated unless and until a new edition is produced. Toolkits that are actively maintained have the potential to stay current, whereas those that are not inevitably degrade over time, although they may continue to be “good enough” for several years. Out of 64 ITD-related toolkits that our team has examined, 16 are not maintained.

Toolkits with ongoing funding vary in the attention paid to maintenance. Replacing broken web links to individual online tools is probably the most straight-forward maintenance task and can be done on an ad-hoc basis, as broken web links are noticed, or through regular systematic checking and replacement. For example, systematic link checking for the Integrated Research Toolkit occurs approximately every six months and takes around three to four hours each time; for the i2Insights blog and repository a similar amount of time is spent on monthly checks. For the Shape-ID Toolkit, the founding consortium created an Editorial Board with former partners to support the longevity of the toolkit. Links are updated whenever these generous volunteers have time because no funds have yet been granted for maintenance.

Replacing or updating outdated resources requires active ongoing engagement with the resources and the broader field to know when resources have become outdated. Occasionally a broken web link will indicate that a tool no longer exists, or an author will notify the toolkit curator that a resource has been updated. But usually those curating the toolkit must use their judgment about whether the resources in the toolkit are still good enough or need refreshing. It can be hard to determine that a tool is out-of-date. Which tools are used and how is highly dependent on the local problem and other aspects of context, as well as the skills and preferences of the tool users. A way around this challenge is to leave judgments about tool utility to the users of the toolkit. In the Shape-ID case, the Editorial Board makes those decisions based on the expertise of its members.

How often new resources are added depends on the nature and purpose of the toolkit. Toolkits with a relatively narrow focus, e.g., teamwork or stakeholder engagement, and those which were comprehensive when established, may only occasionally find new tools to add. Toolkits with a wide reach and those that are built up gradually, rather than establishing a core first, may add new resources more frequently.

Toolkit maintenance also involves assessing how well a toolkit is meeting user needs. Assessment can be done indirectly, such as with website statistics, or directly, by asking users. While website statistics are often automatically generated by content management systems (e.g. WordPress), analyzing the statistics takes time, and interpreting what they mean and what actions should be taken can be difficult. Few toolkits publish website statistics, although the i2Insights blog and repository does. Directly asking users about how well a toolkit meets their needs is time-consuming and may be tainted with various sampling and response biases. There is also the risk of raising expectations with users that cannot be met. Occasionally users offer their views about the toolkit without being asked.

Assessment can be done more or less formally, ranging from a multi-faceted evaluation study to spot-checks. Between these two extremes, the i2Insights blog and repository publishes quarterly statistics and an annual review; these reports summarize many usage statistics but do not analyze mixed method indicators of multiple aspects of quality. A more formal evaluation requires so many resources that it may only be worthwhile for summative purposes.

It is rare for outdated online toolkits to be decommissioned; mostly they add to cyberspace junk. As one of us (GB) discovered when decommissioning a toolkit, this process is time-consuming and expensive. Of course, it is possible to simply allow a toolkit to degrade overtime or to shut it down in various ways (eg deleting it or letting the domain name account lapse). Such a ‘scorched earth’ approach does not consider the ‘public good,’ including the effects on users (especially repeat users), search engines which have indexed the toolkit, links from collaborator websites or from the internet at large, let alone the significant investment of time and energy by those who created and maintained the toolkit and their supporters, including funders. Decommissioning involves moving content to another active website or archiving it, or some of both, none of which are easy or quick, especially if there is a lot of content (see for example, Bammer and Deane 2002–onwards). Things to consider include the following: where will the content be stored, how will it be arranged, how to rebuild the menu structure, linkages, and other associated text, so that users know what is happening and can still access resources, especially those that are being moved rather than archived. Particularly time consuming, and therefore expensive, is that all links to and from the toolkit under the web owner’s control will need to be individually redirected, sometimes with an explanation, so users can continue to access them seamlessly. All in all, to be a good internet citizen, to care about the content presented to users across the internet, and to support the community the content was created for in the first place, requires a commitment to quality that is ongoing, and which extends beyond just creating content and leaving it alone. To do so is time-consuming, resource intensive and expensive, but at the end of the day, necessary.

Many of the tasks involved in maintaining a toolkit are like those involved in creating it. Hence, the toolkit’s governance or decision-making structure is crucial here, too. As when the toolkit was created, governance can help to ensure that there is sufficient expertise and availability to maintain the toolkit. The governance structure may need to evolve as the toolkit leaves the creation phase and moves into maintenance mode.

#### Expertise.

Much of the expertise involved in creating toolkits— especially developing them—will also support maintaining them, so long as the expertise keeps up with the evolving field of knowledge. Rather than repeat the expertise from above, we list here only the additional skills and knowledge needed for toolkit maintenance:

- Technical maintenance skills, especially checking and fixing operations for online toolkits, and for decommissioning toolkits when necessary.
- Ability to identify new tools, particularly knowing where and how to look for emerging tools and methods.
- Ability to identify outdated tools, especially knowing if they have been completely or only partially superseded.
- Assessment expertise, both formative and summative, of individual tools as well as the entire toolkit.

### Toolkit scholarship

#### Process.

As an evolving practice of science, toolkitting is becoming a subject of research in its own right. The inherent challenges of ITD research have led many to acknowledge that more research on ITD dynamics is necessary (e.g., Klein 2021). Toolkitting is one of these under-investigated dynamics.

Scholarly questions about toolkitting can be asked from afar, as researchers from fields such as science and technology studies and philosophy of science observe toolkitting from the outside. They critically inquire on how and why such toolkits are relevant for the scientific domain, analyzing, for instance, the type of collaborations built or the power asymmetries reproduced (e.g., O’Rourke 2017; see also other “outside-in” studies of ITD dynamics such as Felt et al. 2016). Or, questions about toolkitting could be asked from within, as these scholars undertake toolkitting themselves and observe its effects. When studying toolkits “from within,” toolkit scholars take on the responsibilities of toolkit creators, curators, funders, or users as well as those of researchers. They oscillate between developing or using toolkits and analyzing their potentials and effects, as evidenced by this article and Iris van der Tuin’s (2021) essay. Formal approaches to such research can include design science (Peffers et al. 2007) or constructionist research (Kafai and Resnick 1996).

To date, the study of toolkitting has remained an inconspicuous task often performed by ITD researchers who reflect on their own practices, especially when confronted with ITD challenges. Such scholarly reflections on toolkitting tend to be nested within the academic articles published by toolkit creators as they seek to explain the unique contributions of their toolkits. However scholarly or insightful, these reflections have yet to be recognized and developed as a body of research that can propel the practice of toolkitting as a whole. They are fragmented and dispersed in many different types of publications, ranging from working papers to reports for funding agencies (e.g., Fletcher et al. 2021; Studer and Pohl 2023).

The study of toolkitting and the toolkits it produces has the potential to redefine and reinvent methods and conceptual frameworks for evaluating research and designing funding instruments (Vienni-Baptista 2022; Vienni-Baptista et al. 2022). When used to evaluate research, the study of toolkitting can, for example, identify “fake collaborations” among toolkit teams that may be promoted inadvertently by funders or policymakers who encourage the development of toolkits (Conroy 2020; Dai 2020). Ultimately, the study of toolkitting can lead to stronger ITD funding programs, policies, and collaborations.

Another potential for scholarship on ITD toolkitting is to inform basic understanding of how research works when different disciplinary standards and structures meet. Inter- and transdisciplinarity are like an astronomic nebula, permanently positioned at the boundaries of different fields and thus a prime location for studying the birth of new scientific structures, from ephemeral practices (Crowley et al. 2016) to enduring interdisciplines (Klein 2021). In aiding the adaptation of 21st century research to the complex challenges facing the planet, inter- and transdisciplinarity are the place to watch for lessons learned, and toolkitting may provide a rich source of data, especially if toolkits are well designed and maintained.

#### Expertise.

Although scholarship on toolkitting is still nascent, our experience suggests that several forms of expertise will be valuable, including:

- Familiarity with ITD toolkitting practices, including knowledge of the toolkits landscape, the different ITD communities, and the main challenges of toolkitting. Experience in toolkitting is not necessarily a prerequisite, although commitment and involvement in ITD research is advisable.
- Sensitivity to heterogeneity in ITD practices and tools, which involves respecting rather than seeking to overcome differences among tools, toolkits, and contexts for implementation.
- Socio-material perspectives on research practice, with the ability to apply theoretical resources from fields such as science and technology studies, media studies, design studies, constructionist research, and philosophy of science.
- Being well-versed in research methods, including the limitations of some methods for studying toolkitting and the strengths of others such as participant observation and ethnography.
- Analytical competencies, especially being able to break down toolkitting processes into parts while understanding their interactions and potential contributions to the ITD field.
- Reflective skills for those studying toolkitting from within, particularly learning from one’s own experience of toolkitting or that of others to identify lessons that can further toolkit development.
- Critical thinking skills, including the investigation of contingency, causality, and reliability across cases, that will allow inquiry, for example, into the effects that toolkits have in different kinds of ITD research scenarios.
- Ability to differentiate between evaluation and research, in particular noting, communicating, and operating differently when trying to form a *judgment of quality* versus trying to *understand* a toolkitting phenomenon.
- Epistemic awareness and humility, with consciousness of one’s own biases and the potential to be wrong.

Having articulated the processes and expertise involved in three main toolkitting practices (toolkit creation, maintenance, and scholarship), we next discuss strategies for overcoming persistent challenges in toolkitting.

## The future of toolkitting: working smarter not just harder

As described above, toolkitting as metawork can bring benefits to the practice of ITD research and the development of inter- and transdisciplinarity as a field. Toolkitting can make resources more accessible, useful, and rigorous, enhancing ITD research. It also provides evidence of the common challenges the ITD communities face and how common strategies to overcome these have been developed by researchers and practitioners.

On the other hand, toolkitting comes with risks and imperfections. Toolkitting may inadvertently change how we understand the ITD field. Toolkitting requires drawing boundaries around the included tools and labeling them to support sharing with others. It can thus improve research and peer review by codifying and legitimizing new ways of undertaking ITD research. However, the tools may be “too young,” as when the first word on a subject is not the best word. Or, despite initial evidence of transferability, the tools may ultimately only make sense within the complex culture of the original community. Relatedly, it may cause harm to export the tools to other communities through a toolkit if it amounts to epistemic extraction and exploitation of marginalized communities to benefit dominant ones (Alcoff 2022). Moreover, even if the toolkit is respectfully designed, it may still contribute to the problem of fragmentation if it is not shared widely or in language that resonates with other ITD research communities. It can be as though the toolkit does not exist, leading pockets of potential users to re-invent the resources.

Toolkitting as field building is, therefore, worth undertaking carefully while recognizing that it can never be perfect. It requires practical wisdom that pays special attention to the unintended consequences outlined above and to inevitable trade-offs involved in creating best possible resources in the context of real-world limitations. Toolkitting is only possible with a certain amount of power and privilege that gives one access to the knowledge, platform, funding, and relationships that make toolkits and their study possible. In what follows, we pose focused questions to help toolkit specialists handle this power and privilege with care when building the ITD field, including being mindful of language accessibility and advances in decolonizing research. In overcoming fragmentation, which is a major challenge in the toolkits landscape, key roles are played by toolkit creators, curators, scholars, and funders, so they are our focus in the next sections. However, we acknowledge that toolkit use and users remain an important area for future research.

### Guiding questions for toolkit creators and curators.

There is now considerable experience for toolkit creators and curators to draw on—both creators of new toolkits and curators maintaining existing toolkits. The questions below may be helpful not only when a toolkit is developed, but also at regular intervals throughout its life. The questions can also guide funders in evaluating proposals to create toolkits. These are not the only questions to consider, but they center the most important issues.

Questions about contributions to the ITD field include:

- What is the purpose and how does it help build the ITD field?
- What is the theoretical underpinning of your toolkit?
- Who is the target audience? How have you identified what users of your toolkit want and need?
- What does this toolkit provide that does not already exist? How does the toolkit link to and credit what already exists?
- Who created the knowledge you include, and how do they benefit from sharing this knowledge in your toolkit?
- How will you know that your toolkit has outlived its usefulness?

Questions about quality and quality control include:

- What does your toolkit require to be viable? What are the criteria for assessing the quality of the toolkit?
- How are decisions made about which tools to include?
- What makes a tool worthy of being included in your toolkit? Conversely, on what grounds do you reject possible tools?
- To what extent, and how, do you ensure that the tools meet your quality standards?
- How do you decide when a tool is out-of-date and needs refreshing or replacing?
- How do you ensure that your tool descriptions are comprehensible, and the overall toolkit is usable?

Questions about the practicalities of curating a toolkit include:

- How do potential users learn about your toolkit? How do you help users continue to access and use the toolkit?
- What are the day-to-day needs for maintaining your toolkit and can you secure them in an ongoing fashion?
- How is the toolkit funded? Is there a plan for securing ongoing funding?
- If your toolkit outlives its usefulness or is no longer maintained or funded, how will you prevent it from becoming ‘cyberspace junk’ that may inadvertently hide more up-to-date resources?

For those planning new toolkits, these questions can help examine possible implications before committing to developing the toolkit. Addressing the field-building and quality questions allows toolkit creators to place their work in a broader context, as well as assess their goals and how well they are being achieved. The questions on practicalities are a reminder that toolkits are not only intellectual endeavors, but also digital-material objects, subject to the constraints of time and (cyber)space. These practical questions might seem banal but in fact integrate the intellectual questions around goals that drive toolkit creation. In toolkitting, pragmatics are not parasitic, unnecessary hindrances to the intellectual work but rather material and efficient causal factors enabling the ideas to manifest. It is therefore worth studying and reflecting on how these questions are answered.

### Guiding questions for toolkit scholars.

Scholars who study toolkits and toolkitting can play a key role in effective toolkitting. By reflecting systematically upon trends, goals, and processes, toolkit scholars can help toolkit creators, curators, and funders direct their efforts. To state the obvious, it is important for scholars to share their findings with toolkit practitioners and not only other scholars. This ‘circling back’ is facilitated by scholarpractitioners who have built relationships in both communities.

Toolkitting as a subject of research opens myriad questions about how toolkits shape ITD practices generally and ITD expertise specifically. Key scholarly questions about ITD toolkitting include:

- How are boundary choices deliberated, with what results?
- What knowledge from which communities is included in ITD toolkits?
- Who is building, sustaining, and decommissioning toolkits? What social positions and personal attributes help them do this work?
- What power asymmetries are reproduced in the toolkits?
- Can “good” and “bad” examples of toolkit creation, maintenance, and closure be identified?
- How are toolkits influencing the practice of ITD research, including preparatory education?
- What similarities and differences emerge when toolkits are compared?
- How do various toolkit user groups overlap?
- Which toolkits could be helpfully linked, translated, or consolidated?

Further, as with toolkit creation and curation, toolkit scholarship will benefit from reflexivity:

- How does the scholar’s positionality influence how they study toolkits and toolkitting? How can those influences be managed fairly?
- Who is best positioned to judge the value and quality of toolkitting scholarship?

### Guiding questions for toolkit funders.

From a funder perspective, toolkits have two key roles: (1) toolkits as research infrastructure, and (2) toolkits as reviewer resources.

First, excellent toolkits become essential nodes of research infrastructure and thus deserve long-term funding for sustainability and maintenance. To ensure toolkit investments succeed, funders can support the visibility of existing toolkits, encouraging grant applicants to use existing resources to prepare proposals instead of reinventing resources or using low-grade alternatives. Currently, existing resources are shared haphazardly: whether a researcher learns of a toolkit depends more upon the email lists and social media they follow than the quality or utility of the toolkit. Uneven dissemination feeds duplication of tools and toolkits and the fragmentation of the toolkit landscape, which hinders progress in developing inter- and transdisciplinarity and addressing complex societal problems. Funders therefore play a role in providing long-term financial support, in communicating toolkit availability, and in encouraging systematic use of existing toolkits.

Second, well-designed, up-to-date toolkits also indicate the state of the art in ITD research practice, and thus they are resources for reviewers looking to evaluate ITD research proposals—specifically for evaluating the currency and adequacy of the tools ITD projects intend to use.

Overall, the key issue for funders is to assist in overcoming fragmentation and duplication. This is most likely to be achieved if they work with the entire landscape of toolkits, including identifying toolkits with the most promise that can become keystones of ITD research infrastructure. We expand on the landscape of toolkits in the next section. Key questions for funders include the following:

- How can we make applicants aware of existing toolkits?
- How can we support systematic dissemination of existing toolkits?
- How can we incentivize new and existing toolkits to ensure their resources are findable, accessible, interoperable, and reusable, among other Open Science principles (https://www.go-fair.org/fair-principles/)?
- Are there aspects of ITD research that are not yet adequately covered by existing toolkits?
- Which toolkits have shown promise and, with further support, could become keystones of ITD research infrastructure?
- How can we support the ongoing maintenance of these keystone toolkits?

Questions about toolkits as reviewer resources:

- Does the project propose to use tools and methods from a well-designed, up-to-date ITD toolkit? How do they plan to adapt the methods into their context?
- Which toolkits, that the researchers are not aware of, would be useful to the project?
- How can the project contribute to existing toolkits to further develop the ITD field?

When funders are asked to support the creation of a new toolkit, useful questions can be found among those for Toolkit Creators and Curators above.

### The landscape of toolkits and the case for a federated knowledge bank

#### The landscape of toolkits.

Toolkitting smarter as well as harder requires taking stock—that is, surveying the landscape of toolkits that has resulted from the toolkitting status quo. As discussed in the opening sections of this article, our Working Group identified 64 English-language toolkits relevant to inter- and transdisciplinarity, without attempting to be exhaustive. While our Working Group has created an initial overview of this landscape (ITD Alliance Working Group on Toolkits and Methods, 2023), ideally, a full, ongoing inventory would be undertaken by a consortium of ITD researchers, toolkitting scholars, research policy makers, and funders. It will be difficult work that will require dedicated funds, governance, and time to accomplish.

A comprehensive, ongoing inventory of the landscape of toolkits would meet several needs. First, it would support cumulative learning across toolkits. Each toolkit is a unique infrastructure “intervention” yielding different results, like an experiment. Toolkit funders, creators, users, and scholars could more readily discern what works across this field of real-world experiments and where in the landscape unmet needs lie. Second, a landscape inventory would aid toolkit creators, who could avoid duplicating an existing toolkit, and toolkit users, who could more easily find a toolkit for their purposes. Both uses would result in higher quality project proposals that build upon previous funding investments. Third, including a range of toolkits in one inventory would promote a sense of shared ownership. It would work against competition among toolkit creators, who each want *their* toolkit to be acknowledged, used, and elevated. Instead, toolkits would become a common good of all ITD researchers. Fourth, because toolkits are constitutive artifacts of the fields that produce them, the inventory would help document the evolving structure of the ITD landscape: a moving, pulsing “ecology of spatializing practices” that continually defies definition (Klein 2021, p22). This documentation would guide proposal reviewers when seeking indicators of quality across the landscape. At the same time, a landscape inventory would enable a multitude of toolkitting studies that could not only improve toolkitting but also expand fundamental understanding of how science works.

A comprehensive, ongoing inventory is therefore a first step that enables other relational, cultural, and scholarly work needed to overcome the issue of toolkit fragmentation. This additional work could enable a longer-term solution to fragmentation: a federated knowledge bank.

#### The case for a federated knowledge bank.

Tasks we have already outlined for toolkit creators and curators, scholars, and funders would work against fragmentation: toolkit creators can consider their goals and sources carefully, scholars could document the structure and dynamics of toolkit life cycles, and funders could support an ongoing inventory of the landscape of toolkits. It is better to undertake these tasks independently than not at all, but ideally, these tasks would be coordinated. It would take a large toolkitting project to make room for these complex, complementary efforts.

We see opportunity now for such a large project in the form of a global, federated ITD knowledge bank. This knowledge bank would fulfill the vision set out by Bammer and colleagues (2020), who note the broad diversity of knowledge relevant to tackling complex problems through research. In contrast to past calls for a comprehensive compilation of all ITD resources in a single repository (Bammer 2014; O’Rourke 2017), a federated structure —connecting many toolkits through a common platform or protocol—would overcome fragmentation not by subsuming toolkit diversity but by harnessing it. What could this look like?

Novel consortia point the way. A global, federated ITD knowledge bank could be a use case for the Open Knowledge Network (Baru et al. 2022). The Open Knowledge Network, led by multiple US agencies, aligns with the Next Generation Repositories initiative led by the international Confederation of Open Access Repositories. The Next Generation Repositories effort aims “to position repositories as the foundation for a distributed, globally networked infrastructure for scholarly communication” (Rodrigues et al. 2018, p2). Likewise, “[t]he [Open Knowledge Network] is envisioned as an ethical, trustworthy network of interconnected knowledge graphs” (Baru et al. 2022, p14). Both the Next Generation Repositories and Open Knowledge Network efforts recognize that technical interoperability is key to distributed yet connected public knowledge. Toolkit specialists will note that personal and organizational relationships are, in turn, key to technical interoperability. As the Open Knowledge Network Roadmap asserts, “Successful creation of the [Open Knowledge Network] is much more a sociotechnical challenge… than merely a technical exercise” (Baru et al. 2022, p15).

That is, as a socio-material practice, toolkitting is at once personal and technical, intellectual and pragmatic, normative and descriptive, cause and effect. Toolkitting thus faces interlocking challenges that must be addressed together as research infrastructure development. We suggest that the current state of ITD toolkits and toolkitting—with multiple developments that are not yet entrenched—makes a federated knowledge bank an ideal use case for a networked, open access repository. However, the risks of codifying and colonizing knowledge that apply to individual toolkits also apply to a federated collection of toolkits. For example, if this were an instance of the Open Knowledge Network, funding by multiple US agencies could restrict the issues considered and the languages used, with a likely focus on English language only. In addition to guiding individual toolkit development, the questions above could guide this collective effort in a deliberative process with diverse stakeholders to reach the best possible outcome with the available resources.

## Conclusion

In this article we invite readers to join us in lifting our gaze from individual toolkits (important though these are) to the wider toolkits landscape, the challenges of fragmentation therein, and the necessity of closer attention to toolkitting in advancing inter- and transdisciplinarity. The aim is to raise standards of rigor in ITD research without requiring conformity. How can we best help researchers access the growing number of toolkits, and how can we help toolkits provide the most up-to-date and high-quality set of methods, processes, concepts, heuristics, frameworks, and other resources for designing and implementing ITD research? Not addressing toolkit fragmentation relegates researchers to repeatedly reinventing existing methods, being stymied by the same problems, using low-grade techniques or concepts when more sophisticated ones are available, and generally failing to advance or improve the way complex problems are addressed. A lot of expertise is simply lost—forgotten, unused, and unrecognized by relevant users.

We have proposed three key ideas: (1) increased attention to toolkitting expertise and its strengthening; (2) development of a comprehensive, ongoing inventory as a first step in overcoming toolkit fragmentation; and (3) using the inventory as the foundation for a federated knowledge bank. Our study is a first step in advancing the topic and recommending ways to move forward by connecting and developing the range of existing and future toolkits. The approach taken here is not exhaustive and builds from a small number of cases. Its value lies in the richness of the selected cases and the extensive experience the authors bring as toolkit creators and users, and as members of evaluation panels. This work can be extended in the future to connect those involved in ITD toolkitting, bringing together creators and curators, users, funders, and scholars in a shared project that draws on their expertise and resources to achieve a step-change in enhancing ITD research.

## Figures and Tables

**Table 1:** Four current ITD toolkits that some of the authors have been involved in developing, listed in chronological order of development.

Toolkit Name	URL, launch date	Host organization, funding (authors involved)
td-net Toolbox	https://www.transdisciplinarity.ch/toolbox Launched: 2014	Network for Transdisciplinary Research (td-net), Swiss Academies of Arts and Sciences (a+) Funder: multiple grants from the Mercator Foundation Switzerland (Sibylle Studer and Theres Paulsen)
Integration & Implementation Insights (i2Insights) blog and repository	https://i2insights.org Launched: 2015	Integration & Implementation Sciences (i2S), National Center for Epidemiology and Population Health, The Australia National University Funder: no specific funding; supported by salary, voluntary work, and miscellaneous income (Gabriele Bammer)
Integrated Research Toolkit	https://integrated.landcareresearch.co.nz/ Launched: 2019	Manaaki Whenua Landcare Research, Aotearoa New Zealand Funder: Manaaki Whenua (Melissa Robson-Williams)
SHAPE-ID Toolkit	https://www.shapeidtoolkit.eu/ Launched: 2021	Designed by University of Edinburgh, and Trinity College Dublin, with contributions from ETH Zurich, ISINNOVA, IBL PAN, and Jack Spaapen. Funder: Horizon 2020 Framework Programme project (period 2019–2021) (Bianca Vienni-Baptista)
